# Stepwise classification of cancer samples using clinical and molecular data

**DOI:** 10.1186/1471-2105-12-422

**Published:** 2011-10-28

**Authors:** Askar Obulkasim, Gerrit A Meijer, Mark A van de Wiel

**Affiliations:** 1 Department of Epidemiology and Biostatistics, VU University Medical Center, Amsterdam, The Netherlands; 2 Department of Pathology, VU University Medical Center, Amsterdam, The Netherlands; 3 Department of Mathematics, VU University, Amsterdam, The Netherlands

## Abstract

**Background:**

Combining clinical and molecular data types may potentially improve prediction accuracy of a classifier. However, currently there is a shortage of effective and efficient statistical and bioinformatic tools for true integrative data analysis. Existing integrative classifiers have two main disadvantages: First, coarse combination may lead to subtle contributions of one data type to be overshadowed by more obvious contributions of the other. Second, the need to measure both data types for all patients may be both unpractical and (cost) inefficient.

**Results:**

We introduce a novel classification method, a stepwise classifier, which takes advantage of the distinct classification power of clinical data and high-dimensional molecular data. We apply classification algorithms to two data types independently, starting with the traditional clinical risk factors. We only turn to relatively expensive molecular data when the uncertainty of prediction result from clinical data exceeds a predefined limit. Experimental results show that our approach is adaptive: the proportion of samples that needs to be re-classified using molecular data depends on how much we expect the predictive accuracy to increase when re-classifying those samples.

**Conclusions:**

Our method renders a more cost-efficient classifier that is at least as good, and sometimes better, than one based on clinical or molecular data alone. Hence our approach is not just a classifier that minimizes a particular loss function. Instead, it aims to be cost-efficient by avoiding molecular tests for a potentially large subgroup of individuals; moreover, for these individuals a test result would be quickly available, which may lead to reduced waiting times (for diagnosis) and hence lower the patients distress. Stepwise classification is implemented in R-package *stepwiseCM *and available at the Bioconductor website.

## Background

Accurate prognosis of relevant cancer-related endpoints, such as relapse, recurrence or metastasis, may lead to more targeted treatment and avoid unnecessary chemotherapy or surgery. One example is breast cancer recurrence. A major clinical problem of breast cancer recurrence is that by the time primary tumor is diagnosed, microscopic metastases may have already occurred. For this, patients at high risk receive more intensive chemotherapy, endocrine or radiotherapy. Yet, the ability to predict metastasis still remains one of the greatest clinical challenges in oncology.

Classifying cancer subtypes with high precision and predicting treatment outcome are intensive research topics. Traditional cancer prognosis relies on a complex and inexact combination of assessment of clinical and histopathological data. These classic approaches, however, may fail when dealing with atypical tumors or morphologically indistinguishable tumor subtypes; most cancers are both clinically and biologically heterogeneous diseases.

Various clinical or pathological factors have been evaluated as prognosis factors. For example, the treatment of cancer is often based on factors such as age, lymph node status, tumor size, etc. Although these factors provide valuable information about the risk of recurrence, they are generally considered to be insufficient to predict individual patient outcomes and determine an individual patients need for systematic adjuvant therapy. Recent advances in biotechnologies allow us to generate various types of molecular data for the same sample, e.g. copy number aberrations as measured by array CGH, mRNA expression, SNPs, methylation, etc. Each of these distinct data types provides one view of the molecular machinery of the cancer cell. Molecular data allows for adding information to the analysis of biological phenotypes. For illustrating our stepwise approach, we assume clinical data to be comparatively easy to collect and cheap, whereas the molecular data is high-dimensional and relatively expensive. This is, however, not a crucial assumption for the method as such. Moreover, our method is partly motivated by the common perception that classification results from clinical data are more stable than those from high-dimensional molecular data.

Although molecular data and clinical covariates are likely to be correlated, they also contain partly independent information. For example, the extent of lymph node metastasis is currently the key predictor of tumor state, aggressiveness and recurrence risk; this prognostic value can until now not be replaced by any type of molecular data [1]. On the other hand, molecular data alone may supercede other non-genomic factors in prognosis, based on refined and improved molecular technologies that improve the capacity to characterize complex oncogenic processes. Combining these complementary pieces of information may be expected to enhance classification accuracy.

So far few methods have been proposed to integrate clinical and molecular data to obtain accurate cancer prognosis. In [2] a way of integrating microarray data and clinical variables using a modular hierarchical model to predict the outcome for diffuse large B-cell lymphoma (DLBCL) is proposed. Separate modules are constructed for microarray and clinical data. The microarray predictor module is formed by a neural network classifier. For the clinical predictor, an existing clinical prognostic model is converted to a Bayesian classifier. The predictions of the two independent modules are combined and fused to a single prediction. In [3] a Bayesian tree-based approach for combining two data types is proposed. At each node of a tree, the collection of metagenes and clinical factors are sampled to determine which function optimally divides the patients at the node. A split is made when significance exceeds a specified level. An integrative approach in which clinical factors combined with gene expression data using the stepwise logistic regression procedure has been introduced in [4]. The logit transformation of the patients 7-year progression-free probability (PFP) calculated from the nomogram is imposed as the first variable and gene variables are added until optimal classification is achieved. In [5] a study on how to quantify the additive accuracy of the prognosis of cancer patients using gene classifiers in addition to clinical characteristics is conducted. In [6] a method which uses partial least squares (PLS) dimension reduction on molecular data and applies the random forest algorithm (RF) on both clinical and reduced molecular data is proposed. In [7] a mixture expert model to combine clinical and gene expression using different functions to incorporate both types of features has been proposed. Different gene selection techniques are applied before applying an integrative mixture expert model. More extensive overviews on integrating clinical and high-dimensional molecular data for the purpose of prediction are available in [8,9].

All these approaches require the presence of molecular data for all patients, which may be costly, impractical or inefficient. Moreover, optimal combination of these low and high-dimensional data are still under debate [5]. The stepwise approach we propose requires molecular data to be available for a subset of patients only. At the same time it aims to achieve high accuracy. Moreover, it applies classification to two types of data independently, thereby eliminating the concern about optimal combination. We illustrate the performance of our methods using three publicly available data sets.

## Method

### What can we gain by the stepwise approach?

**Figure 1:**
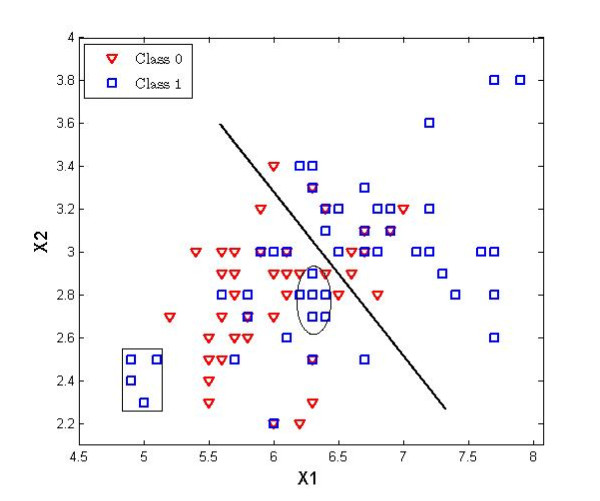
**Illustration of a bad neighborhood concept in the clinical data space using the linear discriminant analysis**. A bad neighborhood is a place in the data space where samples form a cluster on the wrong side of the decision border. It may be located near the decision border (ellipsis) or far from it (rectangle).

We aim to capture the distinct prediction power of each data type in such a way that they are complementary rather than redundant to each other. The 'economic' data type (e.g. standard clinical risk factors) is used in the first stage, while the more expensive data type is only used for user defined fixed proportion of samples whose re-classification scores estimated from the first stage are ranked on the top (descending order). We show that this leads to both accurate and efficient classifiers. The initial motivation for our method was the observation that, whatever algorithm was used, small clusters of samples occurred, which were either misclassified by the clinical classifier or the molecular one, but not by both. This illustrates that the formation of clusters of misclassified samples is not algorithm dependent, but simply a consequence of the potential existence of the subgroups within classes, a well-known phenomenon in complex diseases like cancer. The stepwise approach tries to improve upon the accuracies of the clinical classifier by capturing samples which lie at the wrong side of the decision border. It initially classifies samples using clinical data (see Figure 1) and detects bad neighborhoods (subspaces of the clinical feature space where the classification error is high). Then, it only reclassifies a sample using the molecular data when: 1) it is positioned in a bad neighborhood either close to the decision border (ellipse) or relatively far from it (rectangle), and 2) there is room for an improvement when the sample will be re-classified by molecular data. The second condition is further explained in Section 2.2.3.

#### The stepwise classification procedure

The procedure for the stepwise classification method is as follows:

1. Obtain a prediction label for every sample in the training set using two data types, separately.

2. Calculate a distance matrix for the training set using the two data types independently.

3. Project the test set onto the clinical feature space.

4. Estimate the re-classification score (RS) for each test sample. RS is a combination of the sample's local error rate in the clinical space and score for the potential improvement when re-classifying it.

5. Rank the re-classification score in descending order and reclassify a pre-defined proportion of samples which are ranked on the top with the molecular data classifier.

In the following sections we give a detailed description of each step.

#### The prediction step

In order to assess the performance of each of the two data types, user defined classification algorithm(s) is (are) applied to the two data types independently to obtain the predictions of the training set. This is one of the characteristics of our method. We apply existing algorithms to construct independent prediction models with the training set.

#### The distance metric

Since we try to determine the bad neighborhood in both clinical and molecular data spaces, we need a distance metric which can measure the similarity between samples in the heterogeneous data spaces. High-dimensional molecular data are usually in ratio scale, while clinical data often has continuous, binary and nominal features. So, we are hampered by the distance calculation which is suitable for both types of data. We could discretize the continuous numeric features into the categorical features to make the data homogenous, but this may lead to loss of information [10]. Applying different weighting parameters for the categorical feature and the numerical feature has been proposed [10], but the behavior of the weighting parameter is not yet fully understood and it is difficult to find an optimal one.

Inspired by the work of [11], we present a method that overcomes the aforementioned problems by using the Random Forest (RF) algorithm [12] for calculating similarity (referred to as 'proximity') between samples. Random Forest (a collection of decision trees) is originally introduced for the classification problem, but at this stage we only use it for the distance matrix calculation.

The proximity matrix is a by-product of the tree construction process. For a given forest *ψ*, we compute the proximity between two samples *X*_1 _and *X*_2 _in the following way. For each of the two samples we first propagate their values down all the trees within *ψ*. Next, the terminal node position for each sample in each of the trees is recorded. Let *Z*_1*i *_be the terminal node position of *X*_1 _in the *i^th ^*tree and define *Z*_2*i *_analogously. Then, the proximity between *X*_1 _and *X*_2 _is set to

(1) $$S\left(X_{1},X_{2}\right)=\frac{1}{T}\overset{T}{\underset{i=1}{\sum }}I\left(Z_{1i}==Z_{2i}\right)$$

**Figure 2:**
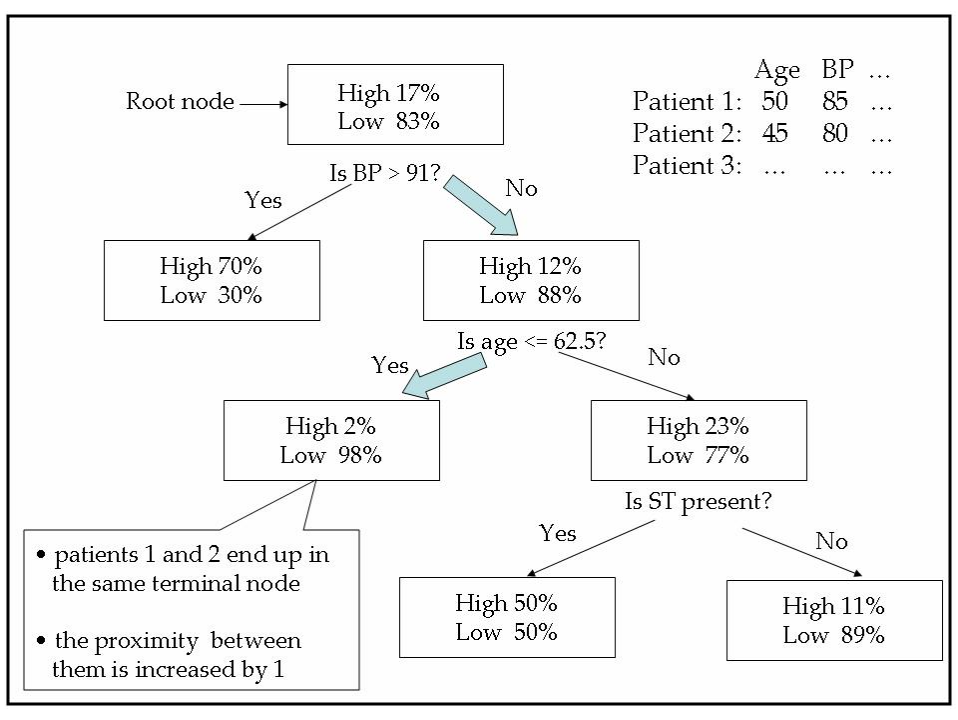
**Illustration of proximity values calculation using the random forest algorithm**.

*T *is the number of trees in *ψ *and *I *is identity function. The intuition is that similar observations should be in the same terminal nodes more often than dissimilar ones. For example, suppose we have a tree as in Figure 2. According to the first splitting criteria (say, *BP *> 91 or *BP *≤ 91) these two patients go to the right node (bold arrow). Second splitting criteria (say, *age *> 62.5 or *age *≤ 62.5) pass them to the left terminal node (bold arrow). Since patient 1 and patient 2 end up in the same terminal node, we increase their proximity value by one. When a test set is present, the proximity of each case in the test set to each case in the training set is also computed. Decision trees and the randomization strategy within the random forest can handle mixed variable types well. Another main reason for using the random forest is their ability to utilize the redundant features (e.g. in molecular data), its invariance to the monotonic transformations of the input variables and its robustness to outlying observations. This is also important in our case, since if a sample has values for one redundant feature but not for others, we can still use this feature for the proximity calculation process. Our method uses both clinical and molecular data separately to construct two random forests from the training set. The resulting forests are used to determine the proximities in both spaces. The proximities from the RF are intrinsic rather than an ad hoc measure [12].

**Figure 3:**
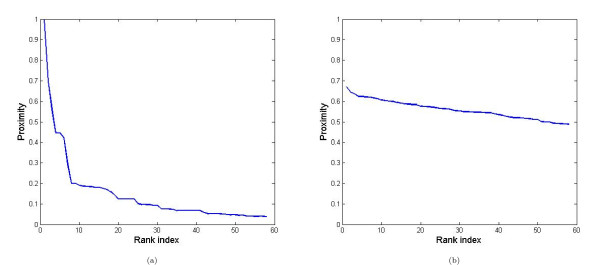
**Proximity values from the clinical data**. Exemplary figure for the proximity values (with respect to Sample 1) using the clinical data (a). X-axis: rank of the proximity values of the other samples w.r.t. Sample 1. Y-axis: values of the proximities. Figure (b) corresponds to the proximity values (with respect to Sample 1) using the molecular data.

Figure 3 illustrate the ranked proximity values of all samples with respect to sample 1, as computed from clinical (a) and molecular data (b), respectively. We notice that the ranges of proximity values are different in the two data types. In the clinical data space, the ranked proximity values decrease quickly and level off around zero. In the molecular data space, however, none of the proximity values are zero. We speculate that this is because of the high dimensionality of molecular data. Similar behavior was observed when the proximities were computed w.r.t. other samples. This difference in proximity value distributions means that direct use of the proximity values may not be appropriate. Therefore, we prefer an approach which only depends on the relative proximity values instead of the numeric values. So, we choose to use the rank index of the proximity value instead of the proximity value itself. We observed that the rank-based approach is usually superior.

Our method starts from the following information: a training set for which true class labels are available, as well as two predicted ones as obtained from user-defined classifiers on clinical and molecular covariates, separately. To correctly estimate the re-classification score of test samples, we aim to efficiently use the information hidden in the two training data sets. Our approach tries to classify the incoming samples with clinical data as often as possible (with reasonable accuracy) and only turns to molecular data when the re-classification score lies below a certain threshold. To this end, for every test sample our approach acknowledges that molecular information is not given. So, the locality information borrowed from two training spaces can not be weighted equally. Based on the work of [13] where the concept of pseudo nearest neighbor is discussed, we introduce the rank-based pseudo nearest neighbor score of the test sample in two data spaces, separately. First, we introduce the pseudo nearest neighbor score in the clinical data space

(2) $$C_{ij}^{R}={ℜ}_{ij}^{R}\times \frac{1}{j},\;C_{ij}^{W}={ℜ}_{ij}^{W}\times \frac{1}{j}$$

where ${ℜ}_{ij}^{R}\left({ℜ}_{ij}^{W}\right)$ denotes the rank of the proximity value between the test sample *i *and its *j^th ^*closest correctly (wrongly) classified neighbor. $C_{ij}^{R}$ and $C_{ij}^{W}$ denote the weighted rank of the *j^th ^*closest neighbor of the test sample *i *from the correctly classified samples group and the incorrectly classified sample group, respectively.

Next, we discuss how to use a similar concept in the molecular space.

**Figure 4:**
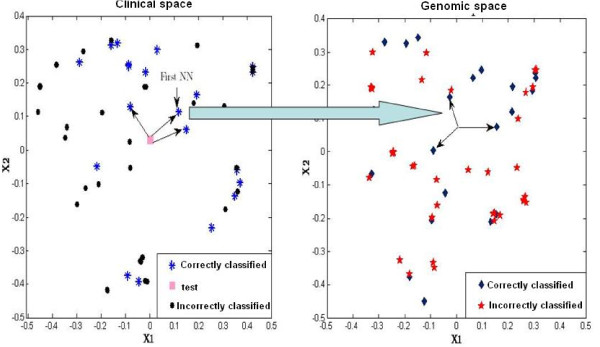
**The indirect mapping (IM)**. For visualization purpose, the IM is shown in the two-dimensional space. Here, *X*1 and *X*2 are the imaginary continuous variables, used for illustrative purpose only.

The main goal of our method is to determine a small group of samples which potentially benefit most by measuring the molecular features. In the stepwise classification approach, we initially do not use the molecular information of a test sample, only use its clinical information. So, we can not directly project the test samples onto the molecular data space to determine its *K *nearest neighbors in the same fashion as in the clinical space. To address this problem, we proposed an indirect mapping (IM) approach called Neighbor's Neighbors. For the sake of simplicity, we illustrate the IM in the two-dimensional space (see Figure 4) where the two *X*1 and *X*2 axis represent the imaginary continuous variables. First project the test sample onto the clinical space to find its *K *nearest neighbors among the training samples, then map these *K *nearest neighbors to the molecular data space one by one to determine their own *K *nearest neighbors. The logic behind this is that, when the two samples have similar clinical characteristics, then they may also share the same molecular characteristics, due to the potential association between the two types of features. E.g. estrogen receptor (ER) status is a well-known prognostic factor in breast cancer and it is also well known that this factor is strongly associated with the genomic features (amplification on the chromosome 17 and over-expression of the HER2-gene). This is further illustrated in additional file 1. So, even if we do not have the genomic information of a test sample, we may approximately locate the position of test sample in the genomic data space based on such correlation. The indirect mapping we introduce tries to take advantage of this correlation and creates a bridge between the two data spaces. We conducted a small simulation study, which indeed illustrates that a stepwise classifier with the indirect mapping may be slightly superior to the one without the indirect mapping when the correlations in the range 0 - 0.5 are present (see additional file 1 for more details). When the correlations are absent, the indirect mapping adds some noise to the RS, equivalent to blind mapping. However, use of a large neighborhood in the molecular space implies that the contribution of the 'random' molecular predictions to the RS is small, because $G_{CR\left(ij\right)}^{R}$ and $G_{CR\left(ij\right)}^{W}$ (see Equations 3 and 4) will be roughly equal and hence $C_{ij}^{R}$ and $C_{ij}^{W}$ will dominate the RS. When the correlations are large, indirect mapping is also less useful (but harmless at the same time), because the molecular information are not likely to change the class assignment for the concerning test samples, and hence the order of the RS remains largely unchanged as well. The weighted rank-based pseudo nearest neighbor in the molecular data space of the *l^th ^*nearest correctly classified neighbor found in the clinical space is defined by

(3) $$G_{l}^{R}=\overset{K}{\underset{t=1}{\sum }}{\tilde{ℜ}}_{lt}\times \frac{1}{t},\;G_{l}^{W}=\overset{K}{\underset{t=1}{\sum }}{\tilde{ℜ}}_{lt}\times \frac{1}{t},G_{l}=G_{l}^{W}-G_{l}^{R}$$

where ${\tilde{ℜ}}_{lt}^{R}$ denote the rank of the proximity value of the sample *l *and its *t^th ^*nearest correctly classified neighbor in the molecular data space. Since *l *is a training sample, we know its class label and hence we include it in the neighborhood space (automatically with rank 1). Positive *G_l _*means that the *l^th ^*sample is located in a region of the molecular data space where the correctly classified samples are closer to it than the incorrectly classified samples. This further implies that sample *l *is a neighbor of the *i^th ^*test sample in the clinical space, the *i^th ^*test sample might fall in this safe region as well. Aggregate the locality information with respect to the *i^th ^*test sample from the two different spaces as follows:

(4) $$Right_{i}=\overset{K}{\underset{j=1}{\sum }}\left[C_{ij}^{R}\times {G_{CR}}_{\left(ij\right)}\right],\;Wrong_{i}=\overset{K}{\underset{j=1}{\sum }}\left[C_{ij}^{W}\times {G_{CW}}_{\left(ij\right)}\right],$$

where *CR*_(*ij*)_(*CW*_(*ij*)_) is the sample index of the *j^th ^*nearest correctly (incorrectly) classified neighbor of the *i^th ^*test sample in the clinical space. *Right_i_*(*Wrong_i_*) denotes the aggregated neighborhood information from correctly (incorrectly) classified neighbors using clinical and molecular data. A large value means that the *i^th ^*test sample is located in a relatively good (bad) neighborhood. Finally, the re-classification score of the *i^th ^*test sample is defined by

(5) $$RS_{i}=Right_{i}-Wrong_{i}$$

The large *RS_i _*means that the aggregated information from the two spaces indicate that the *i^th ^*test sample is likely to benefit more when classified using molecular data. After estimating the *RS *for all test samples, we order these in descending order and only pass the top ranked pre-defined proportion of samples to molecular data for re-classification (see additional file 1 for an example calculation of the RS). In practice, the test samples often arrive one at the time. In such cases, we advise the following implementation of our procedure. First, based on the classification curve, as obtained from the study data, and practical (e.g. cost) considerations decide upon the desired re-classification proportion. Then, this proportion implies a cut point for the RS, which is then used prospectively. If the study data is a good reflection of the entire population, one may expect that this strategy indeed prospectively reclassifies the desired proportion. Naturally, one may monitor the re-classified proportion for the given cut point, and adjust if necessary.

We observe from the *RS *calculation step that there is only one parameter in the whole procedure: the number of nearest neighbors (*K*). We could use cross-validation to find an optimal value for it in the training period. But, to simplify the entire calculation, we eliminate this parameter. We apply the inverse of the neighborhood index as a weight, which suppresses the impact of the proximity values of samples located far from the sample under consideration. Based on this information, we propose to use the fixed *K*, calculated as follows: let *NCW*(*NGW*) be the number of misclassified training samples in the clinical (molecular) data space and define *NCR *(*NGR*) analogously for correct classification, then

K=min(NCW,NCR,NGW,NGR).

In case the data produce a perfect classification result, which would set *K *= 0, we use *K *= 1. For consistency reasons we prefer to use the same number of neighbors for all the 2 * 2 = 4 instances (clinical/molecular; correct/wrong).

## Results

### Data

The stepwise classification method is evaluated on three publicly available real data sets for which both clinical and gene expression data were available. These three data sets have also been analyzed in [7] using the integrative approach. The first data set is a breast cancer data set [14] containing 256 samples, 75 samples with recurrence and 181 without recurrence metastasis within 5 years. It consists of expression levels of 5537 genes. The available clinical variables are age (nominal), number of positive nodes (nominal), tumor size (binary), tumor grade (ordinal), estrogen receptor status (binary), surgery type (binary), chemotherapy treated status (binary), hormonal therapy treated status (binary). The second data set is a central nervous system (CNS) tumor data set [15] which has been used to predict the response of childhood malignant embryonal tumors of CNS to the therapy. The data set is composed of 60 patients, 21 patients died and 39 survived within 24 months. Gene expression data has 7128 genes and clinical features are Chang stage (nominal), sex (binary), age (nominal), chemo Cx (binary), chemo VP (binary). We also evaluated our method on prostate cancer data [4]. Analysis results of this data set are given in additional file 1.

### Algorithms used

For the sake of comparing accuracy and efficiency of our stepwise approach with the fully integrative classifier in the MAclinical R-package [6] we apply the random forest (*RF*) for clinical data and the Plsrf-x (partial least square dimension reduction plus RF) and the Plsrf-x-pv (pre-validated PLS dimension reduction plus RF) for molecular data, separately. Besides that, we also use a variety of well-known classification algorithms, e.g. penalized logistic regression, top scoring pair (TSP) [16] and support vector machine (SVM) (see additional file 1). We use full molecular data without any pre-filtering. To achieve more stable results prediction accuracy is estimated using 10 times 10-fold CV evaluation. Moreover, 5-fold inner-CV is applied to each training set for each classification algorithm for the purpose of parameter tuning.

## Results

Our approach is expected to be most useful when the molecular data classifier has higher classification accuracy than the clinical one. We also present scenarios where clinical data classifier has better performance than molecular data classifier and the scenario where both data sets have equal performances to illustrate how our approach adapts to the situation. Note that the relevant scenario for a given study depends on the data, but also on the pre-specified classification algorithms for both data types. Therefore, we illustrate the three scenarios by combinations of data sets and algorithms that lead to the given scenario. To come to a fair comparison between approaches (clinical, molecular, fully integrated or stepwise) we fix the classification algorithm used on clinical and the one used on molecular data in each illustration.

### The scenario where molecular data classifier performs better than clinical data

**Figure 5:**
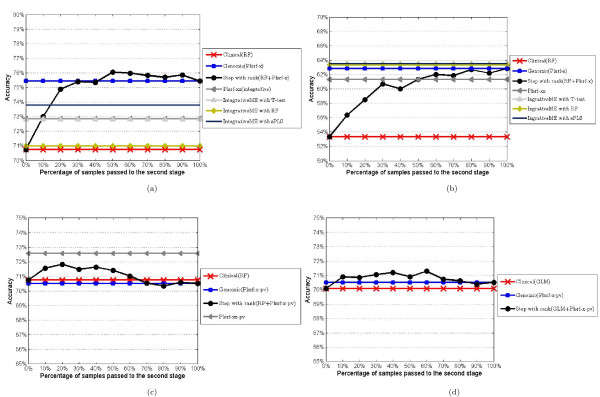
**Classification performances comparison**. Figure (a) shows the classification results for the breast cancer data. The X-axis represents the percentage of samples that are classified using the expression data and the Y-axis represents the corresponding accuracy at that point. For example, the third point from the left in the black curve denotes, 75% accuracy is achieved by classifying 20% percent of samples using the expression data. *Clinical(RF) *denotes the accuracy curve from the *RF *algorithm using the clinical data. *Genomic(Plsrf-x) *denotes the accuracy curve from the *plsrf *using the expression data. *Step with rank(RF+Plsrf-x) *denotes the accuracy curve from our approach. *Plsrf-xz *denotes the accuracy curve from [6] and last three denote to the accuracy curves from [7] with three different feature extraction criteria. Figure (b) shows the results from the CNS cancer data with the same algorithm settings as in (a). Figure (c) shows the result from the breast cancer data when *Plsrf *with *PV *is applied to the expression data; the algorithm used for the clinical remains unchanged. Since the result from IntegrativeME is not available for the *Plsrf *with *PV *setting, here we only compare our approach with the one from [6]. The last Figure (d) corresponds to the result from the breast cancer data. Here *GLM *is applied to the clinical data and the algorithm for the expression data remains unchanged.

As a benchmark, we first calculate the classification accuracy of each data types separately. We apply the RF algorithm on clinical data and the PLS-RF on molecular data. We also show the results from the two fully integrative approaches. First, the integrative mixture expert [7] with three different feature selection techniques and second, the Plsrf-xz [6]. For reasons of comparability with the IntegrativeME methods, we do not use the Plsrf with PV (pre-validation) here. Figure 5a illustrates the performance of the classifiers for the breast cancer data. We observe that none of the integrative classifiers achieves the accuracy close to the one from molecular data alone (75.43%). The result from the IntegrativeME approach with the RF as a feature selection is even worse (close to the clinical classifier accuracy). The accuracies from the rank-based stepwise approach quickly reach the accuracy of the molecular data classifier at 30% of reclassified samples. The highest accuracy is reached when re-classifying 50% samples. Figure 5b illustrates the result from the CNS cancer data. In this data setting, both clinical and the molecular data classifier have very low accuracies (53% and 63%, respectively). All three methods from the IntegrativeME perform better than the molecular data classifier, but the difference is very small (in particular in an absolute sense, given the small sample size). The stepwise classifier attains almost the same accuracy as the purely integrative approaches without fully using molecular data. Hence, in the context of the given classification algorithms, the stepwise classifier is an efficient alternative to the fully integrative classifier for both data sets.

### The scenario where clinical and the molecular data classifier perform equally well

In this scenario, the desired result is that the stepwise classifier produces an accuracy somewhat higher than those of both the molecular and clinical data classifiers. Besides that, the maximum accuracy should be attained without fully using the molecular data. Since the clinical and the molecular data classifiers have equal performance, passing samples to the molecular classifier may help less in terms of accuracy than in the previous scenario. We illustrate this scenario by using the breast cancer data again, but with different classification algorithms. In the first setting we use RF on clinical data and the RF-PLS with pre-validation on molecular data. In the second setting we use GLM on clinical data and the same molecular data classifiers as in the first setting. Here, we did use the plsrf with PV and only compared it with the stepwise approach that includes the PV (the IntegrativeME does not apply in this setting). We prefer to use the PV in this comparison, because, conceptually, it should be useful (see [6,17].) We observe in Figures 5c and 5d that in both settings the stepwise classifier accuracy curves behave as expected.

### The scenario where the clinical data classifier performs better than the molecular data classifier

The results of our approach when the clinical data classifier alone performs better than the molecular data classifier are presented in additional file 1. The optimal result from the stepwise approach in this scenario is high accuracy at the beginning and decreasing accuracy following the increase of the proportion of re-classified samples with molecular data. As expected, the accuracy from the stepwise approach reaches its top at the beginning. Accuracy is close to the one from the IntegrativeME, keeping in mind that the IntegrativeME is a fully integrative approach.

## Discussion

The efficiency gain of the stepwise approach is considerable when the molecular data classifier performs better than clinical data. Our approach nicely adapts to the more powerful data type in an economically efficient manner. After applying the different classification algorithms, we find that when the performances of the two data types are close, the stepwise classification performance is similar to the integrative classifier. If two types of data have unequal performances, then the stepwise approach may outperform the integrative classifier in terms of the accuracy. If the two data type are equally powerful, then a fully integrative approach may outperform our stepwise approach in terms of the accuracy. This is not surprising, because the integrative approaches treat the two data types in a more symmetric way than we do. In the latter case, one should consider whether the gain in accuracy outweighs the loss in efficiency.

The accuracy plots suggest that in many cases it is sufficient to re-classify only a part of the samples. The actual choice of the percentage to be re-classified may depend on the estimated accuracies, but also on the available budget, which might restrict the maximum percentage of samples prospectively re-classified. In such a case several scenarios are possible. Suppose the accuracies of the separate clinical and molecular classifiers are available. If the clinical data classifier is clearly outperforming the molecular data classifier, simply use 0%: no re-classification. If the molecular data classifier is clearly better: use the maximum proportion allowed by the budget or use the percentage where the accuracy curve starts to flatten to save the costs. If the two are competing: take the percentage lower or equal to the maximum that performs best, provided it is allowed by the available budget. We are aware that the reported accuracy rate of the latter procedure might be slightly optimistic (because the best percentage is chosen). However, the bias should be very modest, because it concerns a maximization over very positively correlated quantities: they only differ by the portion of data re-classified.

**Figure 6:**
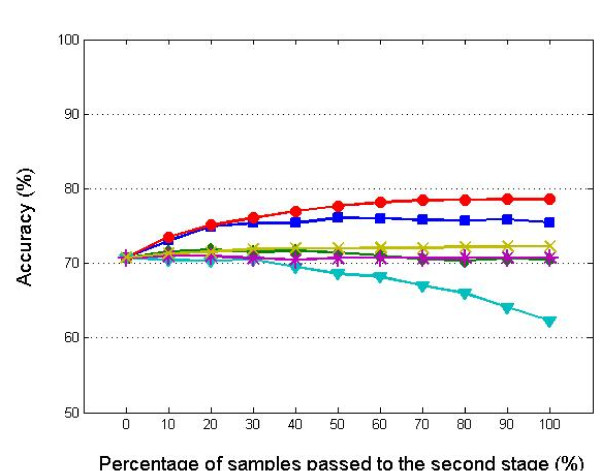
**The stabilization effect (breast cancer data)**. Accuracy curves from the stepwise approach with different algorithms settings. In each curve, the right end corresponds to an accuracy from the expression data and the left end corresponds to an accuracy from the clinical data. The closer to the right end of the curve, the larger the differences in accuracy curves that are observed. This is not unexpected, because closer to the right end more and more samples are classified using the high dimensional expression data and vice versa.

Other attractive properties of our method are adaptivity and stability w.r.t. the classification algorithms used. Figure 6 illustrates the performances of the stepwise approach after applying the different classification algorithms. The left end of each curve corresponds to the accuracy from clinical data alone and the right end corresponds to the accuracy from molecular data alone. As expected, the accuracy fluctuates more and more when we push more samples to the molecular data stage. In each curve there is a point which has the same (or higher) accuracy as the molecular data classifier, but without fully using molecular data and with less variance. It indicates two promising features of our approach. First, for all the six classifiers efficiency can be gained (with respect to 100% re-classification). Even for the best performing molecular classifier SVM, it seems sufficient to re-classify only 60-70%. Second, our method allows to adapt the re-classification percentage to the performance of the molecular data classifier (compare TSP: 0% with SVM 60-70%), leading to more robust (less variable) performance of the entire procedure with respect to the choice of the molecular data classifier as compared to use of a molecular classifier alone. The latter is still true for a fixed percentage like 50%. Note that the robustness with respect to the choice of the classification algorithm is convenient, because optimizing the choice of the algorithm and estimating the accuracy rate of that algorithm using the same data may lead to over-optimism [18,19].

As an alternative to the presented stepwise classifier, we also considered the case where the second stage uses both molecular and clinical data. We experienced that adding clinical data in the second stage may worsen the performance of the stepwise classifier, as illustrated using the case corresponding to Figure 5a (molecular data classifier performs better than clinical one; see additional file 1). The reason for this might be that the stepwise approach passes the sample to the second stage when the sample has relatively high RS. High RS means that the prediction from clinical data for this particular sample is likely to be unreliably. So, adding clinical data to the second stage may do more harm than good in addition to molecular data. We also ran the analysis for the case where molecular and clinical data have equal performances (corresponding to Figure 5d), but no improvement is observed either (result not shown).

## Conclusion

In this paper, we introduce a new classification method which takes advantage of the distinct prediction power of the comparatively cheap traditional clinical risk factors and high-dimensional molecular data. Robust proximity calculation for mixed features and the neighborhood information in the two different data spaces is used to determine a group of samples which are likely to benefit most by measuring and using the comparatively expensive molecular covariates. Our approach not only utilizes the locality information in the clinical data space, but also tries to extract information from the molecular data space by the indirect mapping (IM). We believe that the IM will be useful in the integration field, as it maybe used to quantify the potential benefit of molecular data without actually measuring it. All the calculation steps take place in the clinical data space (for the prospective samples), there is no need to measure the molecular characters for new samples unless sample's re-classification score falls in the user define range. We demonstrated that the stepwise approach may save a considerable amount of samples to be molecularly profiled without losing accuracy. Moreover, our method has the ability to decrease the variation from algorithm to algorithm (adaptivity and stabilization effect). This is a very useful property when one does not have the prior knowledge about the the most suitable algorithm for the data at hand, which is the most common case.

The stepwise classification method fulfills all the criteria for the ideal integrative classifier enumerated in [6]. The self-tuning ability of the stepwise approach deals with different configurations by adjusting itself to the performance of the good data type. How many samples are assigned to which data type depends on the classification performances of the two data types. Our approach aggregates information regarding the classification powers of the two data types and the sample distribution in the two different spaces to compute a re-classification score for every sample. The stepwise approach has the following distinct characters:

1. Efficient while keeping the reasonable classification accuracy

2. Very generally applicable. It is able to work with

• any classification algorithm

• any type of data

The latter implies that our stepwise approach is also applicable when a cheap, standardized molecular platform is available. In such a case, it may be of interest to either reverse the role of the clinical and (cheap) molecular classifier or use the cheap molecular data instead of or in addition to the clinical data in the first stage while keeping the expensive molecular data for the second stage.

One possible drawback of the proposed approach is that the indirect mapping is based on the correlation between clinical and molecular data. If the correlation is very weak, then the indirect mapping does not provide much information. Future work includes the study of more indirect mapping schemes. Another possible extension of our method will be a multi-step approach, where after estimating the re-classification scores in the clinical data space, one is allowed to choose the most optimal data types for re-classification from the available multiple molecular data types.

In short, we develop a flexible and powerful classifier which is based on a multi-objective (cost efficiency and accuracy) formulation of the classification problem. It utilizes the data in a more economical way than other integrative classifiers, while still achieving relatively high accuracy.

## Authors' contributions

AO developed methodology, performed data analysis, wrote the R package and the manuscript. GM conceived this study and critically revised the manuscript. MvdW conceived this study, developed methodology and critically revised the manuscript. All authors read and approved the manuscript.

## Supplementary Material

Additional file 1 **This document provides supplementary information for the calculation of re-classification score, motivation of the indirect mapping and results from our stepwise classifier using different algorithm combinations not included in the paper**. Click here for file
