# Supplementary material for: Stepwise classification of cancer samples using clinical and molecular data
Source: BMC Bioinformatics. 2011 Oct 28;12:422. doi: 10.1186/1471-2105-12-422 (PMC3221726; doi:10.1186/1471-2105-12-422)
Supplement: Additional file 1 — This document provides supplementary information for the calculation of re-classification score, motivation of the indirect mapping and results from our stepwise classifier using different algorithm combinations not included in the paper. [file 1471-2105-12-422-S1.PDF]

Stepwise classification of cancer samples using clinical and molecular data

Additional file

Askar Obulkasim*[,1] Gerrit Meijer[2] Mark van de Wiel[1,3]

[1]Department of Epidemiology and Biostatistics,VU University Medical Center
[2]Department of Pathology,VU University Medical Center
[3]Department of Mathematics,VU University
Amsterdam, The Netherlands

This document provides the supplementary information for calculation of the re-classification score, the motivation for the indirect mapping, the results from the stepwise classifier using the different algorithm combinations not included in the paper and the illustration of the case where the second stage uses both clinical and molecular data.

Does the indirect mapping help?

In order to illustrate the benefit of the indirect mapping, we conducted the following simulation. For the sake of illustration purpose and easy to manipulate the correlation between the two data sets, we use a two dimensional data. We generate 200 random numbers (x_1 and x_2) from the bivariate normal distribution with following parameters:

$$Mean(x_1, x_2) = \begin{pmatrix} 0 \\ 0 \end{pmatrix}$$

$$Cov(x_1, x_2) = \begin{pmatrix} 1 & \rho \times \sigma_1 \times \sigma_2 \\ \rho \times \sigma_1 \times \sigma_2 & 1 \end{pmatrix}.$$

where ρ is the correlation between x_1 and x_2. For each random number we sample ρ from the uniform distribution (0, 0.5). We set this correlation range because we want to illustrate the performance of the indirect mapping in a setting of low correlation between the two data types. In order to represent noisy data types we added independent standard Gaussian noise to both x_1 and x_2. We treat x_1 and x_2 as two different data types and generated the associated binary responses by following way:

$$P_i = \frac{1}{1 + \exp^{-\beta_1 x_1^i}}, \quad i = 1, 2 \ldots, 100, \quad Y_i = Bernoulli(1, P_i).$$

*To whom correspondence should be addressed: PO Box 7057,1007 MB Amsterdam,The Netherlands. Email: askar.wubulikasimu@vumc.nl

$$P_j = \frac{1}{1+\exp^{-\beta_1 x_2^j}}, \quad i = 101, 102 \ldots, 200, \quad Y_j = Bernoulli(1, P_j).$$

We set $\beta_1 = 1.5$ and $\beta_2 = 3$, so that x_2 performs better in discriminating the two classes than x_1 (this parameters setting simulates the scenario in which the molecular data classifier performs better than clinical data). We used x_1 in the first stage and x_2 in the second stage. We applied the logistic regression classifier to both data types. For the distance metric we used Euclidean distance. In order to produce a reliable result, we run the whole simulation with and without the indirect mapping 100 times and take their average. Here, "without the indirect mapping" refers to a stepwise classifier which only uses the clinical space to calculate the re-classification score.

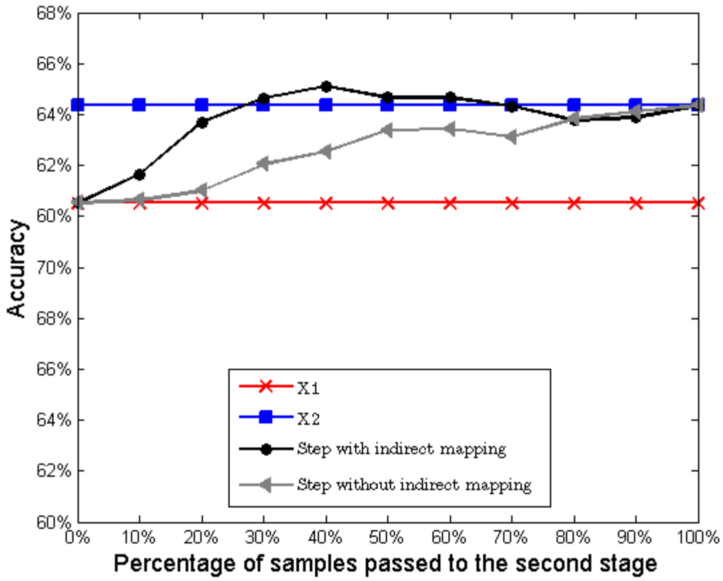

Figure 1: Comparison of the stepwise classifiers in which the accuracies are calculated with and without the indirect mapping

As we observe from the Figure 1, the indirect mapping helps the stepwise classifier to gain some accuracy.

Illustration of correlation between clinical and molecular data

The logic behind the indirect mapping scheme this is that, when two samples have similar clinical characteristics, then they might also share the same molecular characteristics, due to the potential association between the two types of features. Here, we use the estrogen receptor (ER) status in breast cancer data to illustrate this correlation. ER is a well-known prognostic factor in breast cancer and it is also well known that this factor is strongly associated with the genomic features (amplification on the chromosome 17 and over-expression of HER2-gene). To shortly illustrate this once more, we conducted principal component analysis on the molecular data to extract the first principal component (PC). We produced a boxplot using the first PC to observe the samples distribution in the ER positive and ER negative classes. If there is a correlation between the ER

status and the expression profile, then we expect that samples in the two classes have different sample distributions.

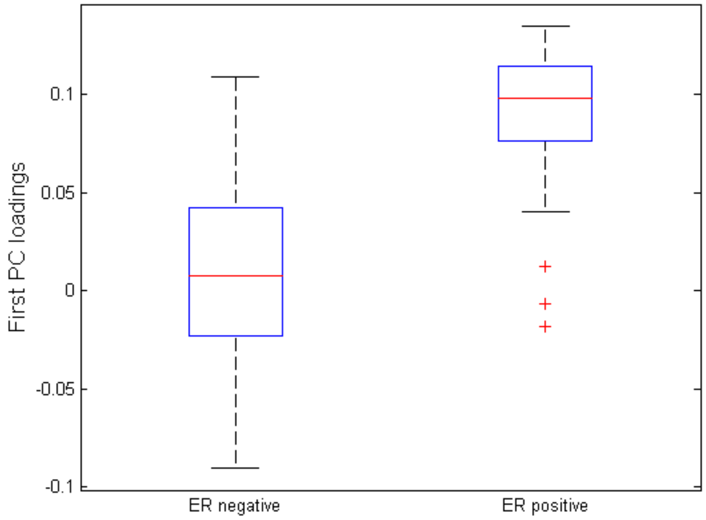

Figure 2: Illustration of correlation between ER status and molecular profile

The difference in the means of the two classes in the Figure 2 shows that correlation exists between the two types of features. This further motivates the use of indirect mapping in our method.

The re-classification score calculation toy example

The Case where a test sample is located in the good neighborhood

Let say we have 10 samples in the training sets (both in clinical and molecular data). After applying the selected classification algorithm to the two data types separately and compare them with their true class labels we have following:

$$Y.cli = (1,0,1,1,0,0,0,0,0,1), \;\; Y.gen = (1,1,1,1,0,1,1,0,1,1),$$

where $Y.cli$ denotes the classification result from clinical data and $Y.gen$ from molecular data. 0 means sample is wrongly classified and 1 means correctly classified. Project a test sample (new sample indicated with index i) onto the clinical data space and measure the proximity between each of those training samples. Let say after ordering (descending) the proximity values, it produces the following order index:

$$order.index^{cli} = (10,3,6,4,1,9,5,2,7,8),$$

use this order index to re-order the $Y.cli$

$$Y.cli.ordered = (1,1,0,1,1,0,0,0,0,0)$$

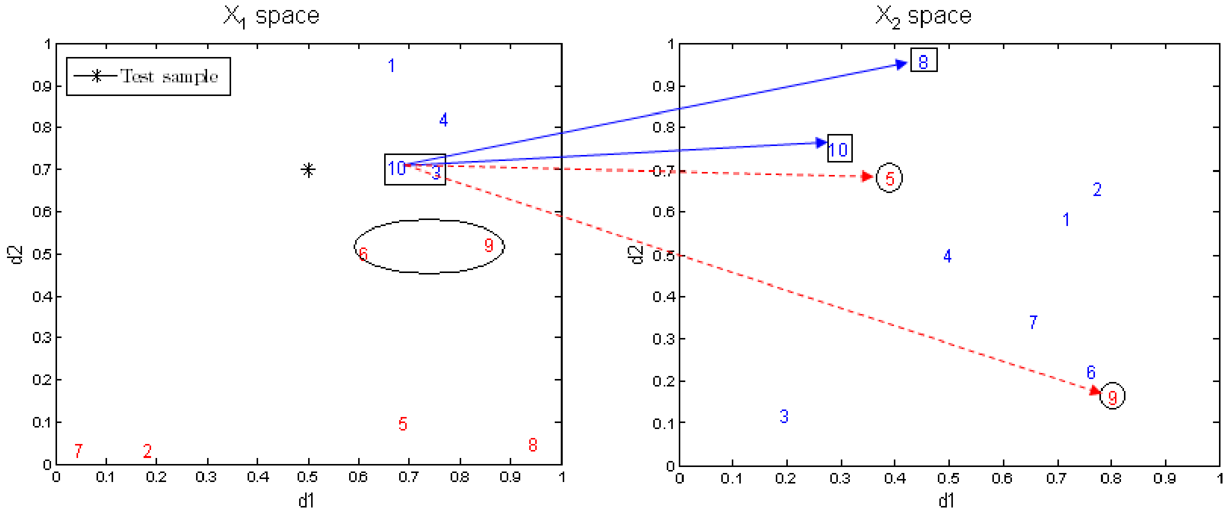

Figure 3: The correctly (10 and 2) and incorrectly (6 and 9) classified neighbors of the test sample in the x_1 space (left). After projecting the first nearest correctly classified neighbor (10) of the test sample onto the x_2 space, we find its nearest correctly classified neighbors (10 and 8) and nearest incorrectly classified neighbors (5 and 9).

Let's take $K = 2$ nearest neighbors from the correctly and the incorrectly classified samples groups, separately. The weighted rank will be

$$C^R_{i1} = 1 \times \frac{1}{1} = 1, \;\; C^R_{i2} = 2 \times \frac{1}{2} = 1$$

In similar way we calculate

$$C^W_{i1} = 3 \times \frac{1}{1} = 3, \;\; C^W_{i2} = 6 \times \frac{1}{2} = 3.$$

the sample indices of the nearest correctly classified $K = 2$ neighbors of the test sample (i) are

$$_{CR(i1)} = 10, \;\; _{CR(i2)} = 3$$

the indices of the incorrectly classified group are

$$_{CW(i1)} = 6, \;\; _{CW(i2)} = 9.$$

Now, we gain the two group of samples indices (for the correctly and incorrectly classified) of the training samples which are close to the test sample (i). In the next step, based on these sample indices we search for the nearest neighbors of them in the molecular data space one by one. Let's consider $_{CR(i1)} = 10$. Let say after ordering (descending) the proximity values with respect to the 10th sample, it produces following order index:

$$order.index^{gen} = (10, 5, 8, 4, 2, 3, 1, 7, 6, 9),$$

use this order index to re-order the $Y.gen$

$$Y.gen.ordered = (1, 0, 1, 1, 1, 1, 1, 1, 1, 0)$$

based on this we calculate

$$G^R_{10} = 1 \times \frac{1}{1} + 3 \times \frac{1}{2} = 2.5,\ \ G^W_{10} = 2 \times \frac{1}{1} + 10 \times \frac{1}{2} = 7,\ \ G_{10}(G_{CR(i1)}) = 7 - 2.5 = 4.5.$$

Let say in similar way we calculate

$$G_3(G_{CR(i2)}) = 3,\ \ G_6(G_{CW(i1)}) = 1.5,\ \ G_9(G_{CW(i2)}) = 5.$$

The final aggregated information for the ith test sample will be

$$Right_i = 1 \times 4.5 + 1 \times 3 = 7.5, \quad Wrong_i = 3 \times 1.5 + 3 \times 5 = 19.5.$$

As a result, the re-classification score of this test sample is

$$RS_i = 7.5 - 19.5 = -12$$

The Case where a test sample is located in the bad neighborhood

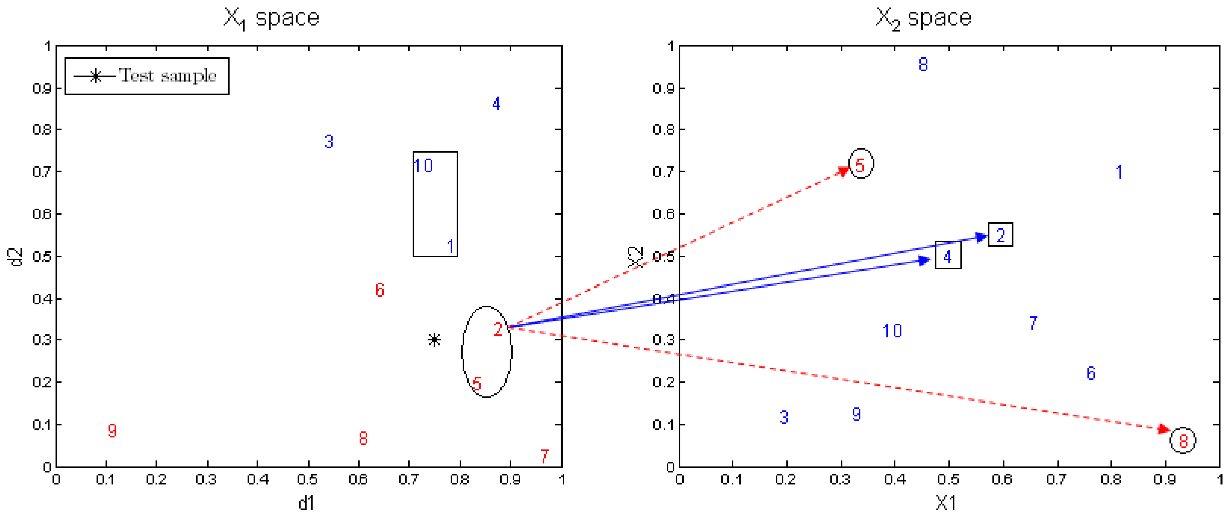

Figure 4: The correctly (10 and 1) and incorrectly (2 and 5) classified neighbors of the test sample in the x_1 space (left). After projecting the first nearest incorrectly classified neighbor (2) of the test sample onto the x_2 space, we find its nearest correctly classified neighbors (4 and 2) and nearest incorrectly classified neighbors (5 and 8).

Let say we have the same settings as in the previous case, after projecting a test sample (new sample indicated with index j) onto the clinical data space and measure the proximity between each of those training samples it produces the following order index:

$$order.index^{cli} = (5, 2, 6, 1, 7, 8, 10, 3, 4, 9),$$

use this order index to re-order the $Y.cli$

$$Y.cli.ordered = (0,0,0,1,0,0,1,1,1,0)$$

Let's take $K=2$ nearest neighbors from the correctly and the incorrectly classified samples groups, separately. The weighted rank will be

$$C^R_{j1} = 4 \times \frac{1}{1} = 4, \;\; C^R_{j2} = 7 \times \frac{1}{2} = 3.5$$

In similar way we calculate

$$C^W_{j1} = 1 \times \frac{1}{1} = 1, \;\; C^W_{j2} = 2 \times \frac{1}{2} = 1.$$

The sample indices of the nearest correctly classified $K=2$ neighbors of the test sample (j) are

$$_{CR(j1)} = 1, \;\; _{CR(j2)} = 10$$

the indices of the incorrectly classified group are

$$_{CW(j1)} = 5, \;\; _{CW(j2)} = 2.$$

Let's consider $_{CR(i1)} = 1$. Let say after ordering (descending) the proximity values with respect to the 1th sample, it produces following order index:

$$order.index^{gen} = (4,2,7,10,5,6,9,1,3,8),$$

use this order index to re-order the $Y.gen$

$$Y.gen.ordered = (1,1,1,1,0,1,1,1,1,0)$$

based on this we calculate

$$G^R_{10} = 1 \times \frac{1}{1} + 2 \times \frac{1}{2} = 2, \;\; G^W_{10} = 5 \times \frac{1}{1} + 10 \times \frac{1}{2} = 10, \;\; G_{10}(G_{CR(i1)}) = 10 - 2 = 8.$$

Let say in similar way we calculate

$$G_3(G_{CR(j2)}) = 5, \;\; G_6(G_{CW(j1)}) = 1.5, \;\; G_9(G_{CW(j2)}) = 3.$$

The final aggregated information for the jth test sample will be

$$Right_j = 4 \times 8 + 3.5 \times 5 = 49.5, \quad Wrong_j = 1 \times 1.5 + 1 \times 3 = 4.5.$$

As a result, the re-classification score of this test sample is

$$RS_j = 49.5 - 4.5 = 45$$

We observe that the re-classification score of the test sample j, which is situated in the bad neighborhood in the x_1 data space, is larger than the test sample i , which is situated in the good neighborhood. Hence, our algorithm will prefer the test sample j over the test sample i to classify on the basis of x_2 data.

The case where clinical data performs better than molecular data

Here, we present the case where clinical data performs better than molecular data using prostate cancer data. Prostate cancer data (Stephenson *et al*, 2005) contains 79 samples, 37 with and 42 without recurrent primary prostate tumors. Pre-filtered gene expression data contains 7884 genes and the clinical factors are composed of serum PSA level (nominal), Gleason stage (ordinal), extra capsular extension (nominal), surgical margin (binary), seminal vesicle invasion (binary), lymph node involvement (binary), TNM (nominal), age (nominal).

Figure 5 illustrates the accuracy of the stepwise approach for prostate cancer data. We use the RF for clinical data and the Plsrf-x for molecular data. The IntegrativeME method with sPLS feature selection attain the highest accuracy (76%). The accuracy from the stepwise approach is somewhat lower than the one from the IntegrativeME, keeping in mind that the IntegrativeME requires 100% molecular data to achieve this. Next, we apply the Plsrf-x-pv to molecular data. Since we do not have the classification result with this algorithm from the IntegrativeME, we only compare with the result from the Plsrf-xz-pv. As we observe from the Figure 2*b*, result from the Plsrf-xz-pv is almost the same as the one from clinical data. The stepwise approach reaches its climax at the beginning as it should and its accuracy is comparative.

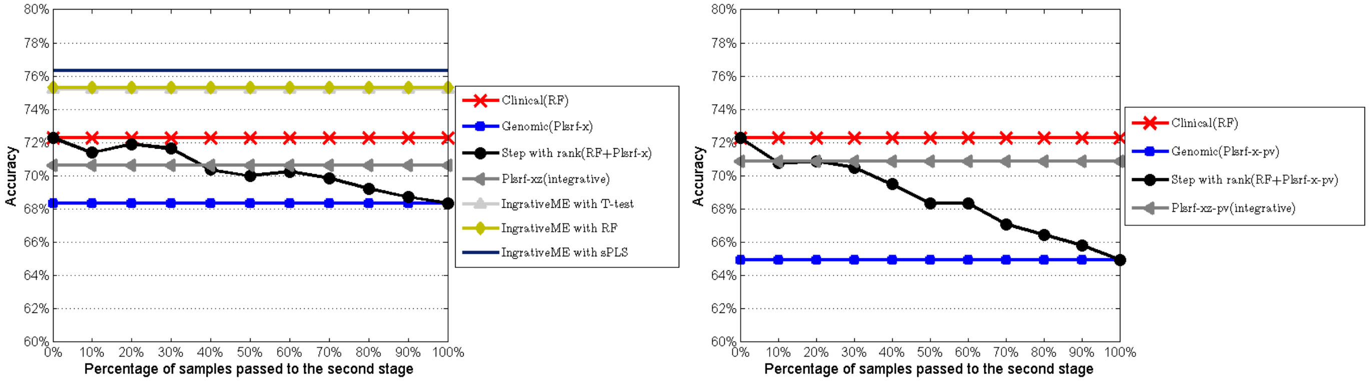

(a) The prostate cancer data

(b) The prostate cancer data

Figure 5: The stepwise classification in which clinical better than molecular data

The results from the different algorithm combinations

In the following part, we illustrate the results of the stepwise approach on the three data sets with different algorithm combinations.

Breast cancer data

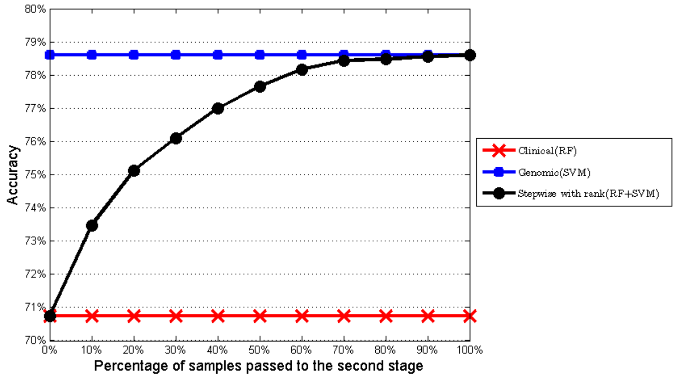

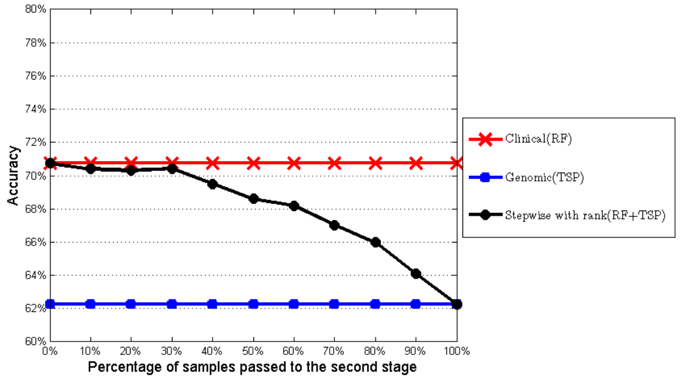

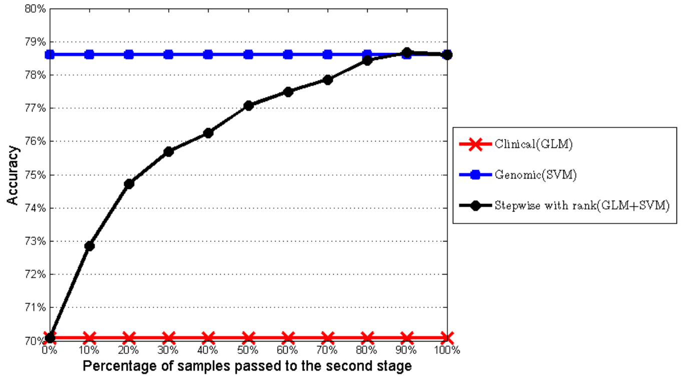

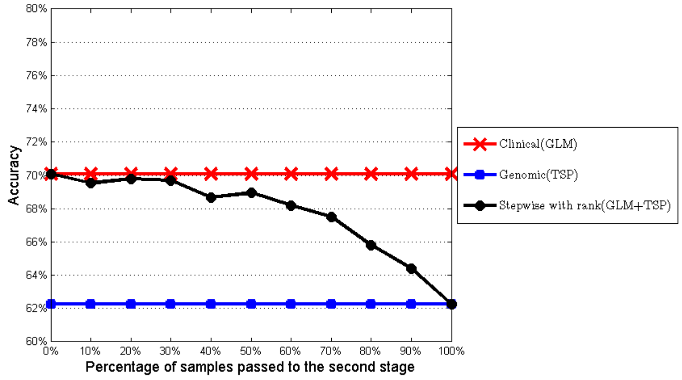

Prostate cancer data

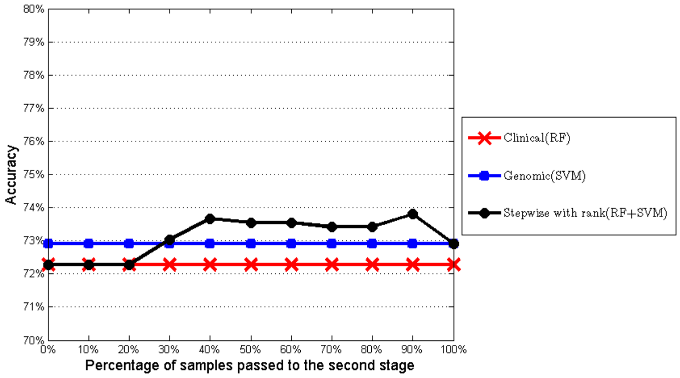

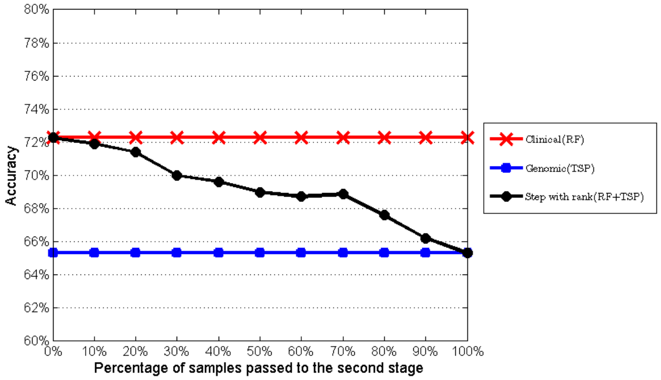

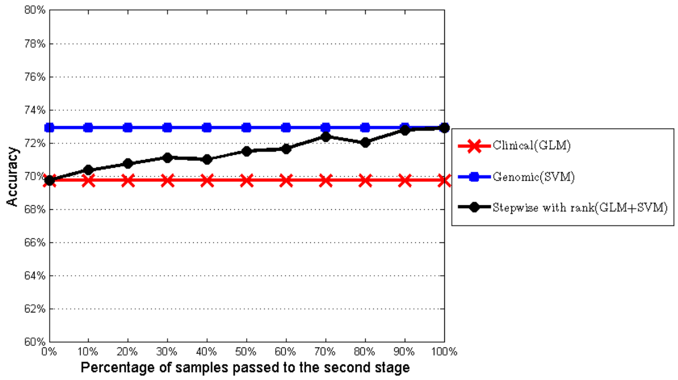

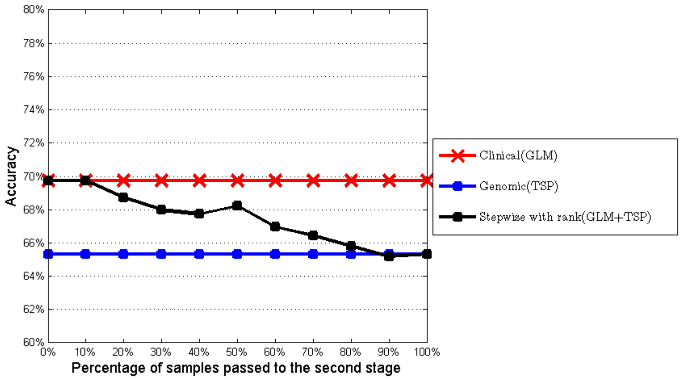

CNS cancer data

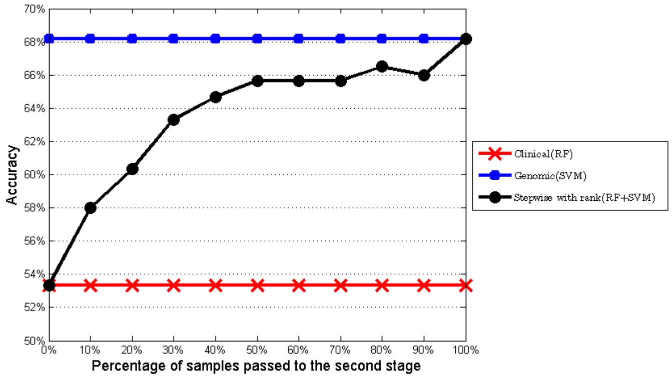

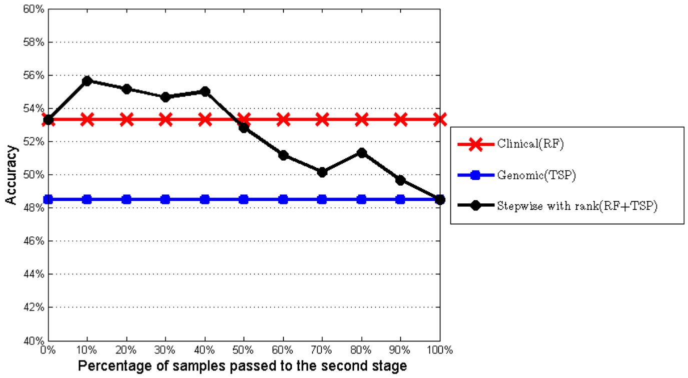

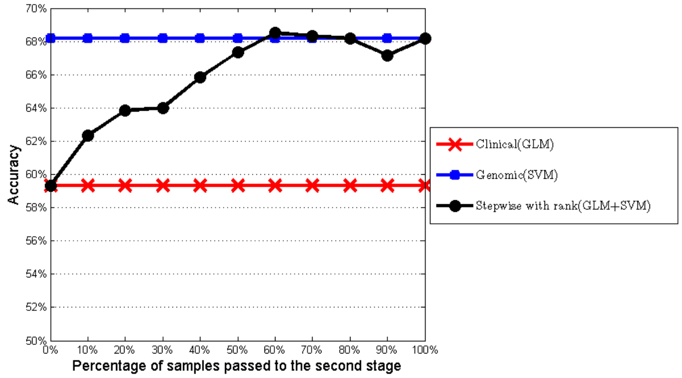

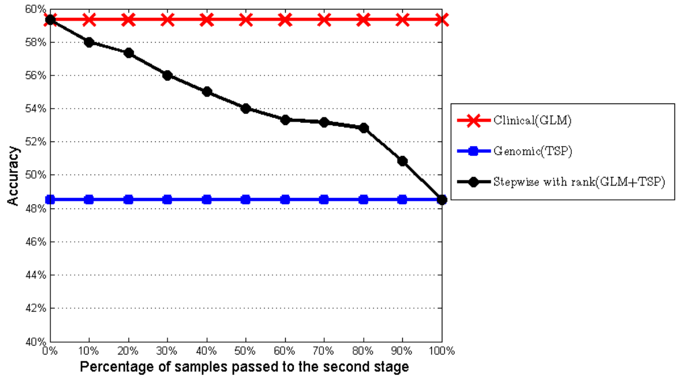

The case where the second stage uses both clinical and molecular data

This part corresponding to the argument ,whether the inclusion of clinical data in the second stage enhance the performance of the stepwise approach, in the main paper. We illustrated this with breast cancer data set, where molecular data performance better than clinical data (corresponding to the Figure 1 in the main paper). We applied the RF for clinical data and applied the combined approach Plsrf-xz to molecular data.

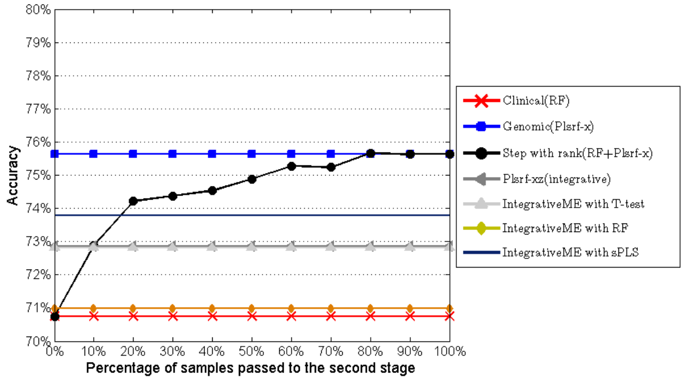

(m) second stage with clinical data

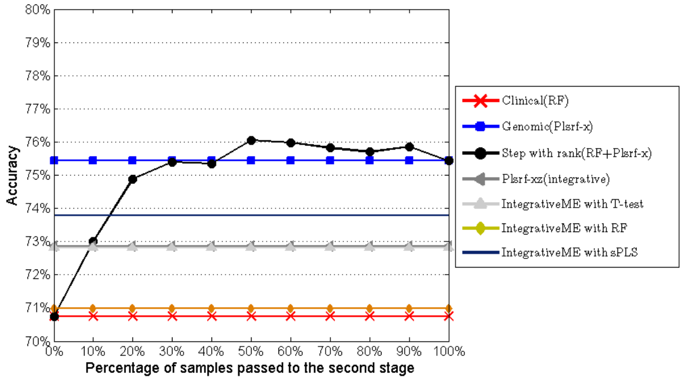

(n) second stage without clinical data

Figure 6: Comparison of the performances of the stepwise approach when the second stage includes and excludes clinical data in addition to molecular data.

As we observe from the Figure 6, inclusion of clinical data into the second stage deteriorates the performance of the stepwise approach.
